# The taphonomic effects of long-term burial in the South African Highveld

**DOI:** 10.1007/s00414-024-03235-x

**Published:** 2024-04-23

**Authors:** Claudia Landsman, Jolandie Myburgh, Anja Meyer

**Affiliations:** 1 Human Variation and Identification Research Unit, School of Anatomical Sciences, Faculty of Health Sciences, University of the Witwatersrand, 7 York Road, Parktown, Johannesburg, 2193 South Africa; 2 Forensic Anthropology Research Centre, Department of Anatomy, School of Medicine, Faculty of Health Sciences, University of Pretoria, Bophelo Road, Prinshof 349-Jr, Pretoria, 0084 South Africa

**Keywords:** South Africa, Highveld, Buried skeletons, Pigs, Taphonomy

## Abstract

Taphonomy studies the environmental effects on remains from the time of deposition to the time of recovery and has been integrated into the field of forensic anthropology. The changes to skeletal remains are dependent on the method of disposal and the surrounding environment. This study focused on buried remains where the type and chemical composition of the soil and the microorganisms present need to be considered. The aim was to investigate the type, frequency, and correlations of the taphonomic alterations of buried domestic pigs*.* Six taphonomic alterations were observed which included depositional staining, adipocere formation, bone weathering, acidic soil corrosion, and plant, and animal activity. Depositional staining, weathering and plant activity were the most common alterations followed by adipocere which was present on 92.3% of the remains. The bones were mostly stained dark brown and brown; however, the trunk region was the only region to present with black staining. The right sides were darker than the left due to the body positioning as most pigs were placed on their right sides and thus were in direct contact with the cadaver decomposition island. Additionally, the right sides presented with more adipocere as well as increased plant activity suggesting that the soil retained water. Darker stains were correlated with a more complete skeleton as adipocere provides some protection. The study confirms that there are various complicated relationships between different taphonomic alterations. A good understanding of them is needed in forensic anthropology to assist in reconstructing the events that occur after death.

**Supplementary Information:**

The online version contains supplementary material available at 10.1007/s00414-024-03235-x.

## Introduction

Taphonomy has become an integral part of forensic anthropological analyses and its incorporation has since broadened the goals of the forensic anthropologist which now includes research that aims to understand the postmortem alterations to soft tissue and skeletal remains such as reconstructing the original position, location, and orientation of a body, and finally to determine the role of human and/or animal involvement with the remains [1–3]. Such information enables anthropologists to potentially estimate the time-since-death or postmortem interval (PMI) in different environments [2, 4–6] and given different scenarios [7–9].

The breakdown of skeletal remains is a highly complex process as many co-dependent variables can influence the appearance of bone. In a natural environmental setting, the surface alterations of bone will differ depending on the nature of disposal. On the surface, solar radiation, temperature, and scavengers have the most impact. This is in contrast to the burial environment where the most influential factors are the different chemical properties of the soil such as pH, groundwater movement, presence of plant roots, the type of soil, availability of oxygen and the presence and types of microorganisms [10–16]. Additionally, the taphonomy of a bone can occasionally be indistinguishable from peri-mortem trauma [8]. For this reason, a greater understanding of the various affecting factors and how each contributes to the breakdown of bone becomes essential in reconstructing peri- and post-mortem events.

Burial environments, like other methods of body disposal (e.g., surface, fluvial environments), expose remains to unique conditions that result in specific decomposition rates [17–21], taphonomic alterations [15, 22–26] and changes to the immediate environment [27–32]. Various studies have analysed the influence of the burial environment on decomposing remains, focusing on the changes to the microstructure of bone [13, 33–35], soil chemistry [36–38] and the microorganisms involved [10, 39].

Furthermore, research on the macroscopic taphonomical changes to the bone in a burial environment remains mostly limited to studies focused on archaeological bone [7, 22, 24, 40–42]. Much of these studies have built upon the pioneering work of Behrensmeyer’s [43] and Gallow et al*.’*s [4]. However, their scoring criteria and stage descriptions are less effective in capturing and describing all the taphonomic alterations seen in buried skeletal remains since both methods are meant to be used for surface scatter remains [25]. It has been suggested that future research focus on a better understanding of decomposition in various micro-environments, including burial environments [24, 25, 42].

Most taphonomic studies that have been done within a South African context have analysed the soft tissue decomposition rates of both buried and surface remains, assessing the effects of clothing and the types of scavengers in the South Africa Highveld [6, 9, 44–49]. Results from these studies have showcased the importance of region-specific data given the influence of different climatic and ecological conditions on the rate of decomposition. Unfortunately, these studies did not include an analysis of skeletonized remains and primarily focused on soft tissue decomposition. Decomposition can, however, reach the extremes of skeletonization if the rate of decomposition is accelerated by environmental factors such as temperature or if the body is only recovered after an extended period [16, 50].

It is, therefore, also necessary to understand the various alterations that may occur post-skeletonization. To date, no South African studies, looking at the rate and effects of burial on post-skeletonized remains in a medico-legal context, could be found. The aim of this investigation was, therefore, to study the type, degree, and frequency of taphonomic alterations and the interrelated relationships between them on a sample of buried skeletonized domestic pigs (*Sus scrofa domesticus*) in the South African Highveld.

## Materials and methods

This study was conducted at the Forensic Anthropology Body Farm on the Miertjie le Roux Experimental Farm, Cullinan District, Gauteng Province (25˚47′20.2’’S; 28˚32′34.3’’E) which belongs to the Faculty of Natural and Agricultural Sciences of the University of Pretoria. The farm is located on the central Highveld plateau of South Africa. The average temperature of the area, for the six years of interment (2014 – 2021) of the remains, ranges between 26.19 and 8.93 degrees Celsius during the summer and winter months, respectively. The area falls into a summer rainfall region (September to April) with a total yearly precipitation of approximately 390.17 – 600 mm. The weather information was obtained from the South African Weather Services from the Bronkhorstspruit weather station.

The vegetation in the area is a combination of grassland and savanna and is thus often referred to as the Bankenveld type or “false grassland” [51] as well as the Rand Highveld grassland [52]. The plant species consists of sour grassland and low sour shrubland [52]. The soil of the area is shallow and rocky and consists of mostly quartzite and shale [53, 54]. This is further confirmed by a geohydrological study undertaken on the Miertjie le Roux Experimental Farm with results indicating a high concentration of quartzite which is typically seen in soil types that have poor drainage given the low porosity of quartzite [55].

The current sample consisted of 39 *Sus scrofa domesticus* (domestic pigs) that died of natural causes between 2014 and 2015 and were buried 24 hours after their death as part of a separate study conducted by Marais-Werner et al*.* [46]. The graves were shallow as the average depth of the graves was 0.75 m and each grave was separated by 3 m to prevent any cross-contamination. Ethical clearance to use and transport the remains was obtained from the University of Witwatersrand Animal Research Ethics Committee (2020/06/07/O) and the University of Pretoria (543/2020). Excavations started and were completed in 2021 where each grave was excavated using standard archaeological techniques [54, 56, 57] with the systematic excavation of soil layers at 20 cm intervals. The remains were exposed, observed and documented in situ before the skeleton was fully removed from the grave for further lab analyses.

### Taphonomic analysis

The taphonomic scoring of soft tissue decomposition is scored and staged using the total body score (TBS) system which notes the stage of decomposition and its associated postmortem interval (PMI) using morphological characteristics of the carcass [2, 6]. The TBS scores three regions (head and neck, trunk and limbs) separately as each region decomposes at a different rate [2]. However, the TBS could not be applied to the skeletal remains as the progression of bone breakdown is not sequential and is heavily dependent on the environmental conditions rather than the time lapsed [2]. For this reason, the only aspect of the TBS system that was incorporated in this study was the use of region-specific staging, which includes the head and neck, trunk and limbs [2]. Initial observation of the remains indicated the presence of six taphonomic alterations which included depositional staining, adipocere formation, bone weathering, acidic soil corrosion, plant activity and animal activity. The presence and degree of each of these six taphonomic alterations were staged for each of the TBS regions (Table 1). To get the overall TBS for each carcass, the most common stage for each taphonomic alteration from each region was used.

**Table 1:** Macroscopic taphonomy stages

	1	2	3	4	5	6
Skeletal completeness	Present and complete	Present and fragmentary	Present and unfused	Present, unfused and fragmentary	Postmortem absent	
Depositional staining	Black	Dark brown	Brown	Yellowish-brown	Yellowish grey	Greyish brown
Adipocere formation	Absent	Coverage of less than half of the region	Coverage on more than half of the region			
Weathering	No weathering and the bone was intact with a smooth surface. This stage was not observed on any of the remains	Rough bone surfaces as cortical bone begins to break down with delamination of the cortical bone. “Marbling” of the bone surface may be present	Weathering penetrates the inner cavities with partial exposure of the trabecular bone and longitudinal cracking may be present	Extensive exposure of the trabecular bone which is beginning to break down	Bone is extremely fragile and fragmented	Complete disintegration/bone shadow, which was noted during excavation
Acidic soil corrosion	Absent	Partial– porous appearance on less than half of the bone	Extensive – porous appearance on more than half of the bone. “Windowing may be present”			
Plant activity	Absent	Root etching/staining	Present with partial macroscopic damage	Present with extensive macroscopic damage to the extent of complete destruction		
Animal activity	Absent	Present				

### Skeletal inventory

A full skeletal inventory was recorded to establish the skeletal completeness as well as the general preservation of the remains. Each bone was staged as being absent or staged into one of five categories when present (Table 1). A percentage of skeletal completeness was estimated to represent overall preservation. This required a total bone count per skeleton which was simplified by counting the skull as one element and excluding epiphyses in the overall count. The sample was comprised of juvenile pigs and thus multiple unfused epiphyses were not included in the overall count. Rather the presence and degree of completeness of the diaphysis were used to record the completeness and general preservation of the limbs. This was done to account for unnecessary inflation caused by individually fragmented or missing skeletal elements. To calculate the percentage completeness of each pig the total skeletal count was divided by 202 as an average adult pig skeleton has 202 skeletal elements [58].

### Depositional staining

The standard analysis for soil colour is the Munsell®’s Soil Colour Chart [59–61]. This chart uses three values to describe a colour which are the hue, value and chroma [61]. A Munsell Color Chart app [version 1.0.1.1] that was developed by KGSc and is available for free on the Google Play Store for Android was used to stage the skeletal remains. The only hues observed in this sample were 7.5 YR, 10YR, 2.5 YR and 5Y. Unfortunately, there are no associations between the Munsell® Soil Colour Chart’s common colour names and the Munsell Color Chart application. Thus, for this study, and in an attempt to simplify the scoring and capturing of the data the following colour categories were grouped; black, dark brown, brown, yellowish-brown, yellowish-grey and greyish-brown and were assigned a stage from one to six (Table 1). Each colour category had a corresponding range of colours and codes unique to the Munsell Soil Color Chart application. These categories and the associated colour chips from the Munsell Soil Color Chart application were established by the investigators as they were the most common colours observed in this sample. Each skeletal region was staged with an overall colour from one of the six categories based on the most prominent colour seen on the bones of a particular region. For example, if the left humerus and radius were mostly dark brown then the entire region was categorised as dark brown. The TBS was then established using the most common colour throughout the regions.

### Adipocere formation

There are no existing scoring methods available to document adipocere formation on skeletal remains and thus a three-stage scoring system was developed for this study. Adipocere is a wax-like substance that can form during cadaver decomposition. It can appear greyish-white on both soft tissues and the bone [36, 62]. Adipocere in this sample was noted as a yellowish-grey colouring on the bone and was staged into one of three categories (Table 1). Each region is made up of multiple skeletal elements, for example, the forelimb is made up of the humerus, radius, ulna, carpals, metacarpals, and phalanges. Each bone can develop adipocere and thus each bone was assessed individually. A collective stage was then assigned to each of the regions. For example, a stage of 1 would be given if there was no evidence of adipocere formation on the bone and if only the humerus had evidence of partial adipocere formation a stage of 2 was given as the humerus makes up less than half of the region’s skeletal elements. A stage of 3 was provided if there was excessive adipocere formation present on multiple bones of that region.

### Bone weathering and acidic soil corrosion

Bone weathering includes the chemical and mechanical changes to the bone which often leads to the bone’s physical destruction. This can be observed as bone-cracking, warping or general erosion (destruction of a bone’s surface layers) [23]. Bone weathering was staged into one of six categories following a modification of Behrensmeyer’s [43] and Ross and Cunningham’s [15] scoring criteria. Behrensmeyer’s [43] stages are the standard for scoring the subaerial weathering of skeletal remains [63] however, these stages do not apply to buried remains [25, 42]. Ross and Cunningham [15] did not observe Behrensmeyer’s stages in their buried sample and thus they created their own stages for their sample. These stages were still not fully applicable to this study either as the stages were associated with long PMIs of up to 30 years. Therefore, amended descriptions from Ross and Cunningham [15], containing more detailed descriptions of this pig sample, were used for the purposes of this study. Behrensmeyer [43] staged the bones using the most extensive stages that were present on more than 1 cm^2^ of bone. In the current study, all the bones of a region were analysed and given a stage between 1 to 6 and the highest value was used to stage the entire region. To establish the TBS per pig the most common stage between the regions was used. The scoring criteria can be seen in Table 1.

Acidic soil may corrode bone, giving it a pitted and porous appearance [23]. The main characteristics of acidic soil corrosion are described by Pokines and Baker [23] and were used as the basis for the scoring method used in this study (Table 1). TBS per pig was established using the most common stage.

### Plant and animal activity

Roots often affect bone surfaces in the form of root staining and etching and may even penetrate the cortical bone to enable growth into the trabecular bone [23]. Plant activity was staged into one of four categories. These were created from descriptions from Pokines and Baker [25] and ranged from absent with a stage of 1 to severe destruction which was designated as a stage of 4 (Table 1).

Any form of animal activity was noted into one of two categories (Table 1). This included any insect activity such as termite damage, scavenger activity (illustrated as bone gnawing), and bioturbation which was determined as present depending on the location of the remains and if they moved from the original margins of the gravesite [64, 65]

### Statistical analysis

The program IBM SPSS version 28 was used to analyse the data. Inter- and intra-observer reliability was conducted using four randomly chosen pigs which were staged by an independent observer or re-staged by the primary observer and the error rates were determined using a weighted Cohen’s Kappa coefficient. Spearman rank correlation coefficient tests were run for each TBS stage against each other for the taphonomic alterations as the data was nonparametric.

## Results

### Type, degree, and frequency of taphonomic alterations

Depositional staining, weathering and plant activity were the most common taphonomic alterations as they were observed on all the pigs. Adipocere was also frequently observed with 36 (92.3%) pigs showing signs of adipocere formation. Animal activity was the least observed taphonomic alteration, being present in only four pigs (10.3%), whereas acidic soil corrosion was present in 29 pigs (74.4%).

### Inter- and intra-observer error

The comparison of the taphonomy stages between the two observers had a moderate to substantial agreement as the Kappa-values were above 0.5 (*p* < 0.05) and the values ranged from 0.500 – 0.882. Staining (0.595), adipocere formation (0.694), acidic soil corrosion (0.500) and plant activity (0.524) were moderate, while weathering was excellent (0.882). 

The average percentage of skeletal elements recovered was 43.0%, with the maximum being 68.8% (grave 21) and a minimum of 11.4% (grave 33). The most observed colours were dark brown (41.0%) and brown (46.2%).

**Fig. 1:**
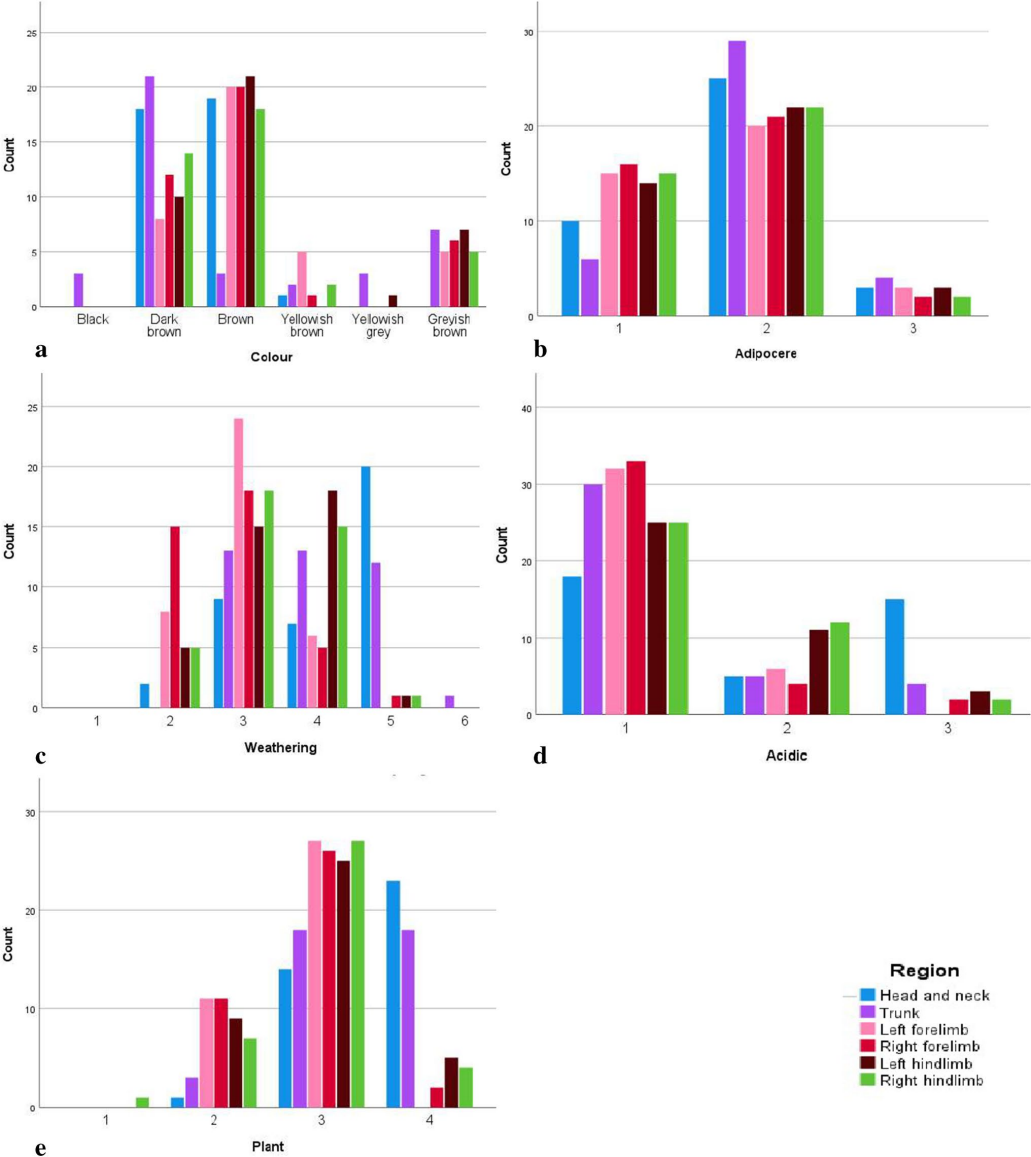
Frequencies of the taphonomic alterations according to the TBS regions

There were distinct differences in soil colour staining between different skeletal regions. The trunk presented with the darkest-coloured soil staining compared to the other regions. The trunk was the only region presenting with black-coloured stains (*n* = 3), with the second most frequent soil colour staining observed being dark brown (*n* = 21). This seems to contrast with the head and neck as well as both the left and right limbs, which were primarily staged as brown. When comparing the left and right sides, the right side was generally darker than the left as the right limbs had more stages for dark brown staining (n forelimbs = 12; n hindlimbs = 14). The left side’s limbs had a higher count for the light brown staining, including brown (n forelimbs = 20: n hindlimbs = 21), yellowish-brown (n forelimbs = 5; n hindlimbs = 0) and greyish brown (n forelimbs = 5; n hindlimbs = 7) (Fig. 1).

**Fig. 2:**
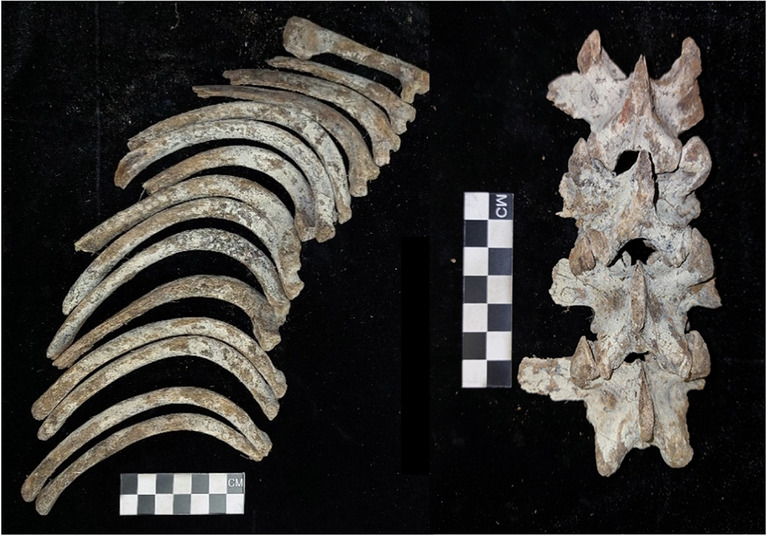
Advanced adipocere on the ribs and vertebrae

**Fig. 3:**
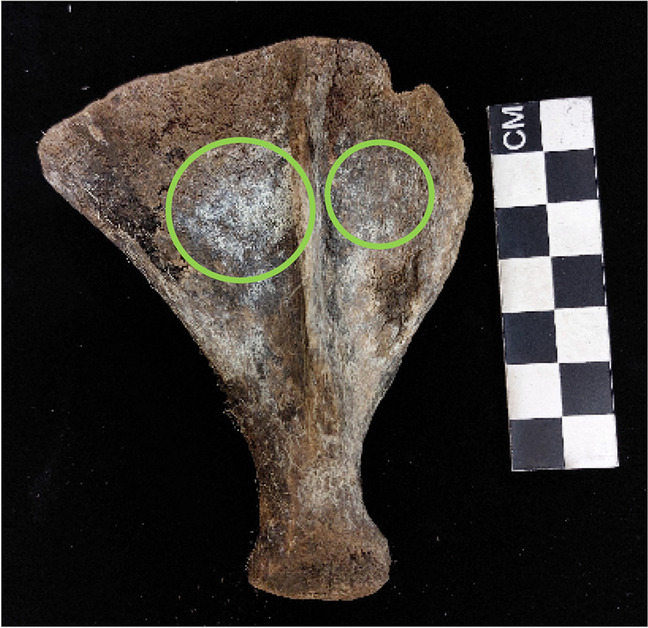
Partial adipocere formation on less than half of the bone (indicated in the green circles)

Adipocere was present on 36 of the pigs with 11 (28.2%) exhibiting advanced adipocere formation (Fig. 2). In assessing adipocere per skeletal region a stage of 2 (59.9%) was mostly obtained which indicates that less than half of the region was covered in adipocere (Fig. 3). Adipocere was most prevalent in the regions of the trunk (*n* = 29 for stage 2; *n* = 4 for stage 3) followed by the head and neck region (*n* = 25 for stage 2; *n* = 3 for stage 3). It is important to note that adipocere also forms within the cranial vault and not only on the external surface of the bone. Additionally, the hindlimbs are more often presented with adipocere when compared to the forelimbs (n right = 22: n left = 22). Stage 2, which represents adipocere on less than half the region, was observed on more of the right limbs (n forelimbs = 21, n hindlimbs = 22) than on the left. However, stage 3, which is advanced adipocere formation, was staged on more of the left limbs than the right (n forelimbs = 3, n hindlimbs = 3).

Weathering was present throughout the sample and was commonly staged as a 3 (53.8% of the sample) which reflects weathering with partial exposure of the trabecular bone. The head and neck were most commonly staged as stage 5, representing bones that are fragile and fragmented (*n* = 20). The area that showed the least amount of weathering (stage 2) was the right forelimbs (*n* = 15). The left side was more weathered than the right side as the left side had a higher count for stages 3 and 4 (n forelimbs = 30; n hindlimbs = 33) compared to the right side. The trunk was the only region that had a stage of 6, representing complete disintegration.

**Fig. 4:**
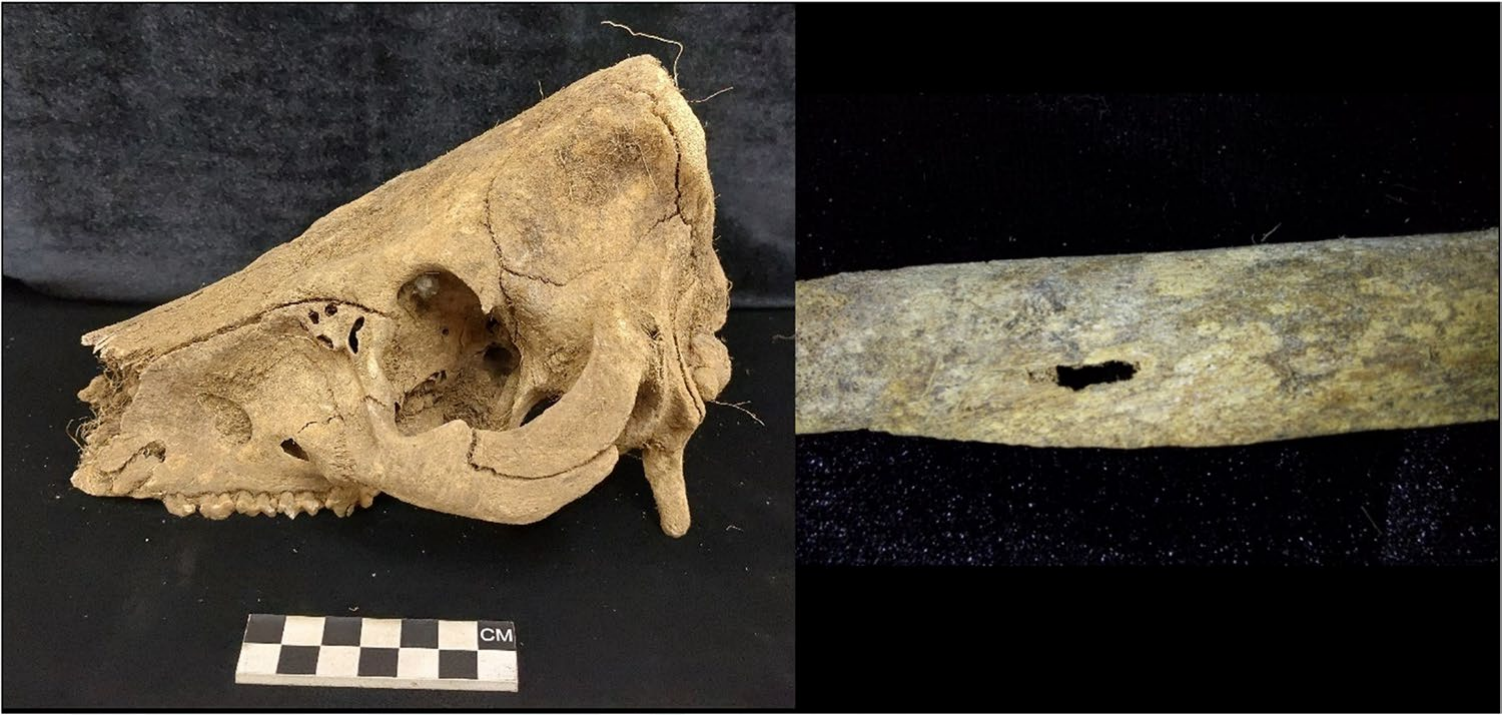
A lacrimal bone and a rib showing “windowing”

Acidic soil corrosion was present on 29 of the pigs. Where present, acidic soil corrosion tended to affect less than half of the skeletal region assessed (18.5%). Acidic soil corrosion was commonly observed on the head and neck as well as the hindlimbs. Corrosion on the head and neck was more extensive as 17 crania (43.6%) were staged as a 3. They were characterised by the presence of windowing, especially on the lacrimal bones (Fig. 4). The hindlimbs are mostly presented as stage 2, which is represented by a scooped and corroded cortical layer that extends into the trabecular bone, of which the femur was most often affected (*n* = 17). The astragali of 12 graves also had a corroded appearance. Additionally, one grave had a rib that showed evidence of “windowing” (Fig. 4) as well as another grave had a circular indentation on the right scapula’s glenoid cavity.

**Fig. 5:**
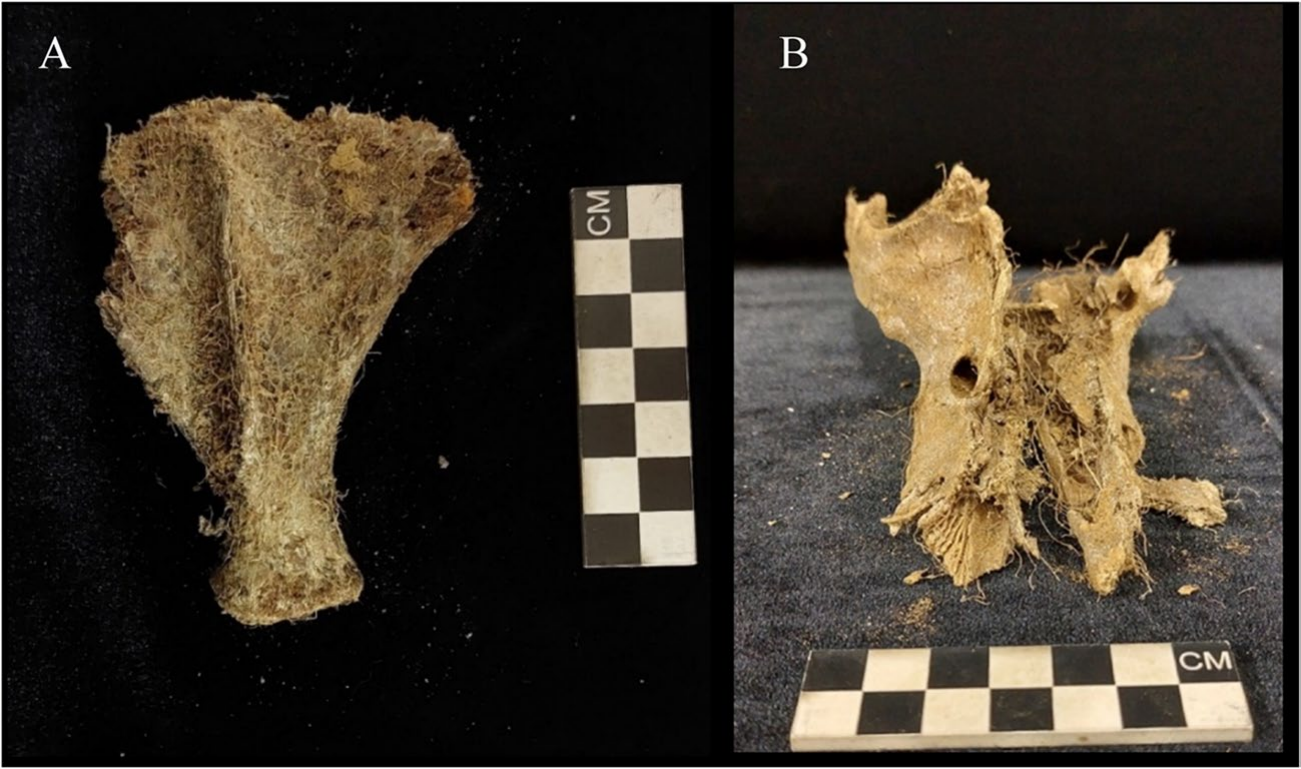
Plant growth causing the destruction of the scapula (**a**) and the skull (**b**)

**Fig. 6:**
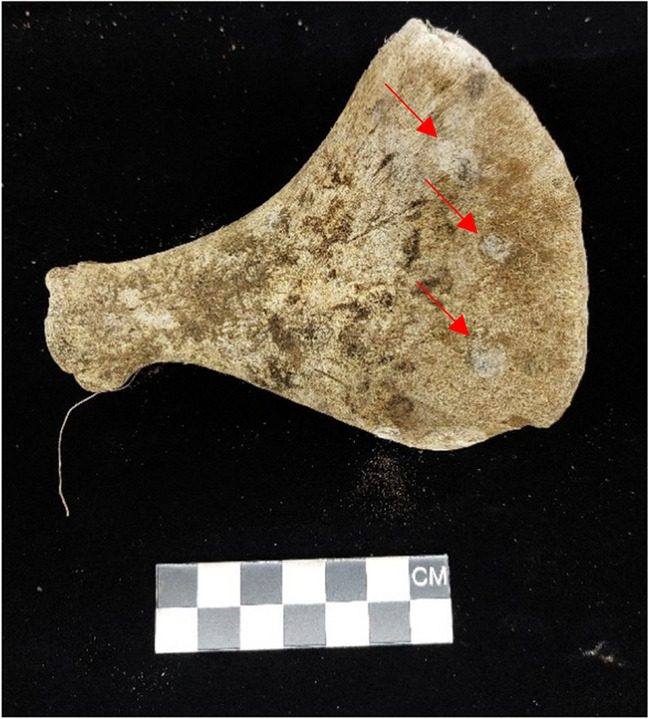
Fungal growth on the scapula (indicated by the red arrows)

All the remains were affected by plant activity (*n* = 39; 100%). Root infiltration causing partial macroscopic damage to the skeleton was present in the majority of the sample (*n* = 28; 71.8%) and skeletal regions (59.1%). Four pigs and 22.4% of the regions had extensive damage caused by plant activity (Fig. 5). Extensive damage was commonly observed on the head and neck region (*n* = 23). Root infiltration into these crania was extensive leading to fracturing along the suture lines and damage to the cortical and trabecular bone, especially around the nasal bones. Additionally, one grave had evidence of possible fungi formation on the left scapula. This was not staged as adipocere since it had a fuzz-like texture. The growths were on top of the cortical bone with a very uniform and circular appearance (Fig. 6).

**Fig. 7:**
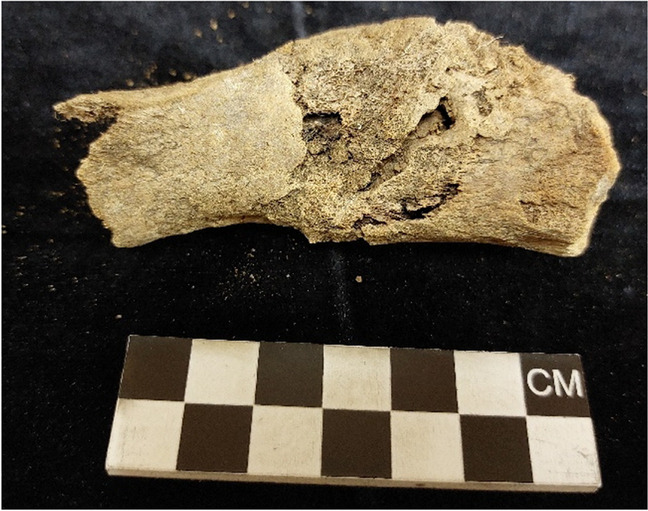
Termite tunnelling through a tibia

Animal activity was primarily absent in the sample (89.7%). There was some insect activity which was identified by the presence of pupa casings from two graves and the tunnel appearance of a tibia on one of these two graves. The tunnel appearance only affected some aspects of the bone, especially the tibial tuberosity. The tunnels ran throughout the bone and there were many entrance and exit holes and thus were excluded as being acidic soil corrosion (Fig. 7).

### Relationships between the taphonomic alterations

Spearman’s rank correlation coefficient analyses were conducted to ascertain whether any correlations between the different taphonomic variables existed. Significant correlations were observed between the completeness of the remains and depositional staining, adipocere formation, weathering, and plant activity (Supplementary material Table 1). Depositional staining had a weak negative correlation with skeletal completeness (rho = -0.151; *p* = 0.021), which suggests that the more complete the skeleton is the darker the depositional staining. Adipocere had a weak positive correlation with skeletal completeness (rho = 0.142; *p* = 0.030). This would indicate that in cases where more adipocere was present, remains were also generally more complete. Both weathering and plant activity had negative correlations with skeletal completeness but the relationship with weathering was stronger (rho = -0.364; *p* < 0.001) than the relationship with plant activity (rho = -0.150; *p* = 0.023). These relationships suggest, as can be expected, that less severe cases of weathering and plant destruction were associated with increased skeletal completeness.

Additionally, depositional staining and acidic soil corrosion presented with a weak negative correlation (rho = -0.161; *p* = 0.014), indicating that decreased levels of soil corrosion were typically seen in remains that presented with lighter depositional staining. A weak positive correlation was also noted between the presence of adipocere formation and plant activity (rho = 0.136; *p* = 0.038). This correlation suggests that as the adipocere formation of a region increases so does the plant activity. Weathering had positive correlations with acidic soil corrosion and plant activity. The relationship between acidic soil corrosion was weaker (rho = 0.141; *p* = 0.032) than that of the plant activity (rho = 0.524; *p* < 0.001). Thus, illustrates that as the weathering stage of a region increased (i.e., the weathering became more severe), the acidic soil corrosion and the plant activity stages also increased.

## Discussion

The average skeletal completeness was low (43.02%) which can be attributed to selective preservation as most of the smaller bones that make up the limbs were not recovered such as the carpals, tarsals, metacarpals and metatarsals, and phalanges. Additionally, small bones are not as well preserved as larger bones since small bones have a larger surface area to volume ratio which increases the rates of reaction between the bone and the surrounding environment causing the bones to degrade faster [16, 66–69].

The most prominent colours observed on the pig skeletons were dark brown (41.0%) and brown (46.2%). Bone interacts with soil through the soil solution which contains soil tannins, minerals, and micro-organisms [70]. The colour of soil, and thus the subsequent bone colour, is indicative of the soil’s composition with regards to the minerals present and the percentage of the organic content [71]. Dark soils are higher in organic content which decomposes to form a black product known as humus.

Differences in the distribution of soil staining could be observed throughout the skeleton. Only the trunk region presented with black (*n* = 3) as well as dark brown (*n* = 21) staining. During soft-tissue decomposition, the trunk region would produce more decomposition fluids compared to the neck/head and limb regions [2, 4] as the trunk contains most of the internal organs that will liquefy during the process of putrefaction and decay. Subsequently, this leads to an influx of carbon and nutrients into the surrounding soils creating a cadaver decomposition island (CDI) that is high in organic content and can stain the soil a darker colour [18, 50, 72]. The CDI can be confirmed by the presence of black soil observed at the level of the carcass from 20 of the graves. The black soil was present after six years of interment which could suggest that these soils do not drain well, and that groundwater movement was limited. The study done by Marais-Werner et al. and others [46, 53–55] suggested that the soil in this area mostly consists of clay, shale and quartzite. Such soils are typically made up of small particles with little porosity, therefore, resulting in decreased water and gas movement [73].

Overall, the right side of the skeleton presented with a darker brown and brown soil staining, compared to the left side. Twenty-six of the right limbs (including both the fore- and hindlimbs) were staged as dark brown. Eighteen trunks also presented with darker staining on the right side. This feature may be associated with the burial position as twenty-seven of the carcasses/pigs were buried on their right side. As most of the right-sided trunks and limbs would have been in direct contact with the CDI it can be assumed that these regions would therefore also be stained a darker soil colour.

Depositional soil staining had a negative relationship with the percent completeness (rho = -0.151; *p* = 0.021). The darker staining is associated with increased organic content of the CDI which is also an environment that is conducive to adipocere formation [36, 72, 74].

Adipocere is a modification of the decomposition process and often occurs in burial environments [36]. The presence of adipocere has been found to decrease the rate of decomposition leading to the preservation of remains [36, 75, 76]. It was noticed that skeletal completeness was increased by the presence of adipocere (rho = 0.142; *p* = 0.030). This would, therefore, account for the increased skeletal completeness in cases where adipocere was present. In this sample, adipocere was present in 92.3% of the graves. It was most prevalent in the regions of the head and neck as well as the trunk. Adipocere was also observed more on the hindlimbs (*n* = 22) and the right sides of the body (*n* = 43). Adipocere formation requires bacteria for hydrolysis of the neutral fats and thus will form in any environment that promotes bacterial survival [75]. The most conducive environment is one that is warm, and anaerobic with moisture such as aquatic and waterlogged environments [36, 77, 78]. Five of the graves during the previous study were waterlogged [79]. During the excavation phase of the current study, there were no waterlogged graves, however, excavations were undertaken during the dry winter months and would, therefore, not necessarily have been waterlogged at the time of excavation. Also, the presence of the decomposing body may have provided sufficient moisture for the formation of adipocere [18, 62]. The head and neck had increased adipocere formation to the extent where adipocere had formed on the internal surface of some of the crania. This could be due to the high fat content of the brain due to the myelin sheaths that surround the neurons [50]. The trunk region has an especially high-fat content that increases the likelihood that adipocere would form in this region. The hindlimbs are in closer contact with the abdomen. The gut also contains the bacteria that initiate decomposition and thus would be present for adipocere formation [31, 50, 79]. However, the grave environment itself may also have contributed to the formation of adipocere. As mentioned previously, the grave soils may not have been well-draining as was indicated by the lack of dark soil stains beyond the level of the carcass. Thus, the remains would have been surrounded by the decomposition fluids for an extended period leading to the formation of adipocere as there would be sufficient moisture and bacteria from the body. An alternative explanation relates to graves acting as water catchment areas as the aerated grave soil (due to backfilling) allows for water movement whereas the sterile soil (representing a clay layer) would prevent the water from seeping down [54, 80].

There was also a positive relationship between adipocere formation and increased plant activity (rho = 0.136; *p* = 0.014). This suggests that graves that presented with adipocere also showed increased plant activity. This would further serve as evidence for the increased moisture retention in these graves which will stimulate plant growth [80].

Adipocere formation was positively correlated with an increase in skeletal completeness per region whereas weathering (rho = -0.364; *p* < 0.001) and plant activity (rho = -0.150; *p* = 0.023) were correlated with a decrease in skeletal preservation. These two taphonomic alterations commonly form part of the diagenesis process as they are typically associated with the destruction of bone [23, 25, 43].

Most of the pigs (*n* = 21; 53.8%) had moderate weathering of the skeletal regions, however, the head and neck, and the trunk were especially fragile and fragmented. The trunk region was the only region that presented with complete disintegration. This was especially true for the skeletons that did not present with adipocere in this region, which would otherwise have slowed down the degradation process. The bones of the trunk are inherently more fragile compared to the rest of the body. Flat bones, such as the ribs, and irregular bones, such as the vertebrae and carpals and tarsals, have thin layers of cortical bone relative to the trabecular bone, which makes them more prone to weathering. These bones also typically have an increased surface area to volume ratio with which the soil solution can react [70]. This will increase the rate of diagenesis and breakdown of the organic and mineral content of the bone and explains why decreased skeletal preservation was observed in these skeletal elements. The bones of the trunk are also exposed more to the organic acids that are produced during decomposition when compared to the other regions [50, 81]. In addition, the left side of the body was more weathered than the right. Again, the right side had more adipocere which would protect it from the weathering process.

Plant activity, such as root infiltration with bone damage, was mostly seen in the same regions as weathering, namely the head, neck and trunk. The roots of plants grow into and around the bone as bone is a good source of nutrients and water [23]. Roots infiltration can be very destructive and cause extensive fracturing of bones [23]. Additionally, plant roots excrete organic acids to enable the uptake of minerals from the bone. In the advanced stages, organic acid secretion can cause damage to the bone that resembles features associated with acidic soil corrosion, such as a roughened cortical surface [23]. In this study, 29 pigs presented with advanced stages of organic secretion which was especially prevalent on the head/neck and hindlimbs.

There was some fungi growth on the scapula from one of the graves, indicating that this grave may have had some oxygen as most fungi are aerobic [82]. This is further supported by the lack of adipocere in this grave which requires an anaerobic environment.

Acidic soil corrosion was common on the head and neck (*n* = 20) as well as on the hindlimbs (*n* = 14). The lacrimal bones of the skull were more often affected which could be due to their thin structure making them more susceptible to damage (*n* = 17). The hindlimbs presented with a scooped appearance on the cortical surface, exposing the trabecular bone, especially on the bones of the femur and astragalus. The proximal femur is more commonly damaged which could be due to it being the most proximal point of the hindlimb and is close to the abdominal region. The abdomen produces substantial organic acids during soft tissue decomposition which may have contributed to the acidic erosion [31, 50]. The hindlimbs are close to the abdomen and thus the large volume of decomposition fluids and organic acids would have also influenced the bones of the hindlimbs. Acidic soil corrosion had a positive relationship with weathering (rho = 0.141, *p* = 0.032) which indicates that as the acidic soil corrosion increased so did weathering. This is not surprising since weathering and acidic soil corrosion have similar destructive characteristics.

Very minimal animal activity was observed in the sample (*n* = 4), but this is expected since burial will significantly reduce access to insects such as flies and scavengers [23]. There was evidence of insect exoskeletons in one of the graves and it was noted during the initial excavation in the previous study, that there was mass colonization of the carcass during the *in-situ* observations [83]. Another grave had evidence of termite tunnels although no termites were observed, and none of the bones presented with termite damage. Termites are often drawn to buried remains as the bones provide them with essentials such as nitrogen and phosphorus [64]. Additionally, the cranium as well as most of the left side of the body from another grave were missing. This grave was especially shallow which may have attracted scavengers.

This study does however present with some limitations. The use of pigs as a proxy for humans is not ideal. Even though pigs have been widely used as a human analogue in decomposition studies as pigs—like humans—are omnivorous, their skin resembles that of humans, they have analogous fat distribution and anatomy, and similar intestinal flora [51–55], they still present with differences in terms of anatomy, osteological composition and differences in gut bacteria [80–82]. These differences may result in different decomposition rates and subsequent taphonomic alterations. This study only presents cross-sectional data and more information on the progression processes may be obtained in future longitudinal studies.

## Conclusion

This study aimed to establish the taphonomic alterations observable on 39 *Sus scrofa domesticus* carcasses buried for six years in the South African Highveld region. Six taphonomic alterations were observed on the skeletal remains including depositional soil staining, adipocere formation, bone weathering, acidic soil corrosion, plant activity and animal activity. There were six different coloured soil stains observed in this sample with dark brown and brown being the most observed. These colours mimic the surrounding matrix and relate to the diagenetic changes of tannins and minerals leaching into bone over time. This study found that areas presenting with darker soil staining generally had an increase skeletal completeness and were often associated with direct contact with the CDI. A large proportion of the sample also presented with adipocere formation, which not only coincided with the darker soil staining observed on bone, but also had a positive correlation with increased skeletal preservation in certain body regions. The low drainage of soils in the Highveld region would have contributed to the formation of adipocere which in turn acted as a buffer between the bone and surrounding environment, resulting in increased skeletal completeness and less bone degradation. The results from this study identified which taphonomic alterations could typically be expected in remains that have been buried for an extended period in the South African Highveld, which to date has not been done for any area in South Africa. In addition, this study also showcased that apart from the influence of regional variation in terms of environmental differences, depositional taphonomy is also dependent on the microenvironmental differences seen between graves in the same region.

### Supplementary Information

Below is the link to the electronic supplementary material.

Supplementary file1 (PDF 179 KB)

## Data Availability

The datasets generated during and/or analysed during the current study are available from the corresponding author on reasonable request.
