# Supplementary material for: The taphonomic effects of long-term burial in the South African Highveld
Source: Int J Legal Med. 2024 Apr 23;138(5):2093–105. doi: 10.1007/s00414-024-03235-x (PMC11306639; doi:10.1007/s00414-024-03235-x)
Supplement: Supplementary file 1 — Supplementary file1 (PDF 179 KB) [file 414_2024_3235_MOESM1_ESM.pdf]

Supplementary material

**Table 1: Region specific inter-taphonomic correlations [n=232]**							
		Completeness	Depositional staining	Adipocere	Weathering	Acidic	Plant
Completeness	Rho						
	Sig.						
Depositional staining	Rho	**-0.151**					
	Sig.	**0.021**					
Adipocere	Rho	**0.142**	0.050				
	Sig.	**0.030**	0.445				
Weathering	Rho	**-0.364**	-0.043	0.033			
	Sig.	**<0.001**	0.518	0.615			
Acidic	Rho	0.074	**-0.161**	-0.024	**0.141**		
	Sig.	0.260	**0.014**	0.716	**0.032**		
Plant	Rho	**-0.150**	0.017	**0.136**	**0.524**	0.017	
	Sig.	**0.023**	0.799	**0.038**	**<0.001**	0.795	
Animal	Rho	0.040	0.000	0.060	0.106	0.018	-0.011
	Sig	0.541	0.994	0.360	0.106	0.779	0.864
